# How to Make a Poultice

**Published:** 1891-11

**Authors:** 


### HOW TO MAKE A POULTICE.

Dr. Brunton, in *Brain*, the new London periodical, gives the following practical hints on this subject : The common practice in making poultices of mixing the linseed meal with hot water and applying it directly to the skin is quite wrong ; because, if we do not wish to burn the patient, we must wait until a great portion of the heat is lost. The proper method is to take a flannel bag (the size of the poultice required), to fill this with linseed poultice as hot as it can possibly be made, and to put between this and the skin a second piece of flannel, so that there shall be at least two thicknesses of flannel between the skin and the poultice itself. Above the poultice should be placed more flannel, or a piece of cotton-wool, to prevcnt it from getting cold. By this method we are able to apply the linseed meal boiling hot, without burning the patient, and the heat gradually diffusing through the flannel affords a grateful sense of relief which cannot be obtained by other means. There are few ways in which such marked relief is given to abdominal pain as by the application of a poultice in this manner.

